# Potential for Introduction of African Swine Fever Virus into High-Biosecurity Pig Farms by Flying Hematophagous Insects

**DOI:** 10.1155/2023/8787621

**Published:** 2023-04-12

**Authors:** Jonno Jorn Stelder, Ann Sofie Olesen, Graham J. Belsham, Thomas Bruun Rasmussen, Anette Bøtner, Lene Jung Kjær, Anette Ella Boklund, René Bødker

**Affiliations:** ^1^Section for Animal Welfare and Disease Control, Department of Veterinary and Animal Sciences, University of Copenhagen, Grønnegårdsvej 8, 1870, Frederiksberg C, Denmark; ^2^Section for Infection Preparedness, Statens Serum Institut, Artillerivej 5, 2300, Copenhagen, Denmark; ^3^Section for Veterinary Clinical Microbiology, Department of Veterinary and Animal Sciences, University of Copenhagen, Stigbøjlen 4, 1870, Frederiksberg C, Denmark

## Abstract

**Background:**

DNA of African swine fever virus (ASFV) has previously been detected in hematophagous insects on ASF outbreak farms. However, it remains unclear whether the viral DNA derived from blood meals that originated from pigs on the outbreak farm or was introduced from infected domestic pigs or wild boar sources located outside of the outbreak farm.

**Methods:**

We caught 644 hematophagous insects on the windows at two non-outbreak high-biosecurity pig farms (i.e., without ASFV-infected pigs) using plastic meshes coated with sticky glue, as well as 3576 hematophagous insects using H-traps on or around the farms. Using PCR analyses, we identified which insects were present, whether these hematophagous insects carried blood from an external (exogenous) source and if the blood contained ASFV DNA.

**Results:**

We found blood meals with ASFV DNA in one pool of five *Haematopota* spp. in the H-traps. From the window traps, we found 0–2.7% of *Haematopota* spp., *Stomoxys calcitrans*, and *Aedes* spp. carrying blood meals from exogenous sources into the farms. Some insects carried bovine blood; the closest registered source for this was 2500m from the pig farm.

**Conclusion:**

Hematophagous insects carrying ASFV-positive blood meals or blood meals from exogenous sources seem to be attracted to high-biosecurity pig farms and attempt to enter them through their windows. Despite the small percentage of insects carrying blood and the small amounts carried by each insect, the large numbers of insects result in a sufficient volume of exogenous blood, potentially containing ASFV, to constitute a non-negligible risk for ASFV-introduction into unprotected pig stables. This study is the first to provide quantitative data on the number of hematophagous insects trying to enter high-biosecurity pig farms. It is also the first to provide information about the origin of their blood meals, indicating that insect-borne introduction of blood containing ASFV into high-biosecurity pig farms is possible and, therefore, could potentially be responsible for some of the outbreaks observed during the summer peaks of infection.

## 1. Introduction

Hematophagous insects are known vectors for a wide variety of pathogens affecting livestock, such as equine infectious anemia virus [1], Rift Valley fever virus [2, 3], Schmallenberg virus [4], and bluetongue virus [5]. One species of hematophagous insect, *Stomoxys calcitrans*, has been shown to be capable of mechanically transmitting African swine fever virus (ASFV) in laboratory settings [6, 7] and hematophagous insects are suspected of transmitting the virus in field settings [8]. ASFV is the cause of a severe hemorrhagic viral disease, termed African swine fever, with a high case fatality rate in domestic pigs and wild boar (*Sus scrofa*) [9]. Since 2014, genotype II ASFV has spread throughout several, mostly eastern, EU countries and has resulted in the culling of millions of domestic pigs [10, 11], causing significant economic losses, despite the many strict biosecurity measures applied to most commercial domestic pig farms to prevent introduction of pathogens.

In the current ASF epidemic observed in the European Union, Lithuania was the first EU-country to report cases in 2014 [12]. Between 2014 and 2016, the annual number of reported cases of ASF amongst wild boar gradually increased (45, 111, and 303, respectively), with a significant increase observed in 2017 (to 1328) [13], followed by a gradual decrease after 2018 (1447) in 2019 (435) and 2020 (158, data until 31^st^ August 2020) [14]. This decline was accompanied by a much reduced wild boar population density [15]. In Lithuanian domestic pigs, an increase in the annual number of outbreaks was observed between 2014 and 2019 (6, 13, 19, 30, and 51 outbreaks in each year, respectively), followed by a substantial decrease in 2019 (19) and 2020 (3, data until 31^st^ August 2020) [14].

For commercial domestic pig farms, a seasonal trend can be observed in the incidence of this disease with a peak of ASF outbreaks during the summer months [8, 12]. This trend coincides with the seasonal activity pattern of hematophagous insects such as mosquitos, biting midges, horseflies, and stable flies [16]. These insects could potentially circumvent the current biosecurity measures at most high-biosecurity pig farms by introducing blood meals containing ASFV through the farm's openings (e.g., windows, open doors, and holes in the roof) after feeding on an infected wild boar or domestic pigs located within the area surrounding the pig farms.

A previous study has shown that ASF outbreaks in domestic pigs, located within a 2 km radius, constituted a significant risk factor for commercial pig farms and backyard farms in Romania [17]. For backyard farms, additional risk factors were found, such as the growth of crops that are attractive to wild boar around the backyard farm and close proximity to cases of ASF in wild boar as well as wild boar abundance [17]. Furthermore, the presence of ASFV positive wild boar around domestic pig farms poses a risk of ASF incidence in domestic pig farms [18]. While airborne transmission of ASF has been confirmed within buildings [19] up to a distance of 2.3 m [20], the practical influence of airborne transmission has not yet been evaluated [19, 20]. Furthermore, it is not expected that airborne spread can explain the spatial risk factor to commercial farms put forth by Boklund et al. [17]. Other factors associated with ASFV transmission, such as professional visits to backyard farms [17], transportation (on trucks), and use of contaminated equipment [21], would generally be mitigated on high-biosecurity pig farms through extensive disinfection and quarantine protocols (for example, see https://ahdb.org.uk/knowledge-library/biosecurity-on-pig-farms). Furthermore, such transmission routes would not be expected to produce the observed seasonal trend in outbreaks.

Well-known transmission routes for ASFV include direct contact [22] and carcasses releasing ASFV into the environment [23] as well as via contaminated materials [7, 22] or feed [17]. Attempts to mitigate these transmission mechanisms are made through the application of strict biosecurity measures. The fact that flying insect-borne mechanical transmission of ASFV can occur has been recognized for decades, and several aspects have been shown as possible in a laboratory setting. Mellor et al. [6] found that *S. calcitrans* can infect domestic pigs with ASFV upon taking a subsequent blood meal on the animals after earlier feeding on viraemic blood. The highest viral load of ASFV can be found in the blood of infected pigs [24, 25], with Olesen et al. [19] describing a realistic titre as 5.8 log_10_ TCID_50_/mL. Olesen et al. [26] found ASFV DNA in the mouthparts for up to 12 h, as well as in the head and body of *S. calcitrans* up to 72 h after feeding on viraemic blood, respectively. They also detected infectious ASFV in bodies of *S. calcitrans* sampled at 3 h and 12 h after feeding. Furthermore, Olesen et al. [7] found that domestic pigs became infected after ingesting *S. calcitrans* that had fed on viraemic blood. These results indicate that, upon entry into a pig farm, there are at least two mechanical transmission pathways for a hematophagous insect carrying blood from an ASFV-infected pig to introduce the virus into a healthy pig inside the farm and cause a new outbreak. A third, and still only theoretical, transmission pathway may be via the squashing of a blood-carrying insect either by pigs, farm workers, or machines. Rupturing of the blood fed insect may contaminate the environment, feed, or water and potentially result in the infection of a pig and thereafter spread directly from the index pig to other pigs in the stable. Infection through contamination inside the farm's environment has already been shown to be possible, for example, through a contaminated pen environment and contaminated swill feed or farm equipment [7, 27].

While mechanical transmission by flying insect vectors of ASFV is possible, field studies on this topic are few and limited. Studies on the flight behaviour of some hematophagous insects indicate that they can fly 100 m [28] or 600 m [29] postfeeding, which might allow them to introduce blood containing ASFV from infected domestic pigs or wild boar within this proximity to a high-biosecurity pig farm. At outbreak farms in Romania, ASFV DNA was found in homogenized pools of both *Culicoides* spp. and *S. calcitrans* caught inside the farm [30]. In Estonia, one *Musca domestica*, one *Drosophila* spp., and two mosquitos that were in close contact with pigs inside an infected unit, tested positive for ASFV DNA [31]. Turčinavičienė et al. [32] reported a higher prevalence of ASFV DNA in invertebrates collected outside outbreak farms compared to non-outbreak farms in Lithuania. Most of these field studies focused on outbreak farms where ASF was present at the time of, or shortly before, the insects were collected. This means that it cannot be determined whether the insects were involved in introduction or transmission, or if they merely picked it up, after an ASF outbreak had already been established. We hypothesize that hematophagous insect vectors could potentially introduce viraemic blood meals into high-biosecurity pig farms through exterior openings, potentially causing a new outbreak of ASF. In order to determine whether hematophagous insects could be the potential source of virus introduction, we set up a quantitative study in Lithuania at two non-outbreak high-biosecurity pig farms. The aims were to: (1) quantify the number of hematophagous insects entering these farms, (2) quantify the percentage of insects carrying blood meals obtained from animals outside of the pig farm premises, (3) quantify the number of hematophagous insects circulating in the immediate area surrounding the stables of these farms, and (4) test whether any blood meals contained ASFV DNA.

## 2. Materials and Methods

To determine which hematophagous insect species fly around high-biosecurity pig farms and attempt to enter the stables, in what numbers they do so, and whether they carry any blood meals or ASFV in the process, we set up two different trapping experiments on each of two Lithuanian pig farms in 2020.

### 2.1. Location and Description of the Two Farms

**Figure 1:**
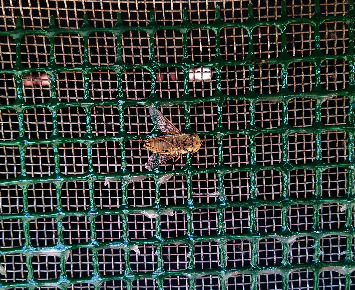
A tabanid caught on the plastic mesh. The original mesh set up by the farm can be seen in the background.

We selected two high-biosecurity pig farms in Lithuania based on convenience and availability (both belong to the same company): farm A in the northern Šiauliai County and farm B in the southwestern Marijampolė County. Farm A is situated in a rural region with a forested area towards the north side. This farm had experienced an outbreak of ASF in 2018. The farm consisted of 10 buildings (or 20 stables) with approximately 18,000 pigs, including 1250 sows. Each stable had a multitude of windows of various sizes. Farm B was mostly surrounded by forest, with agricultural fields to the northeast. The farm consisted of 35 stables, with around 28,000 pigs, including around 1950 sows. Only one stable at farm B had windows. At each farm, we collected insects on windows using sticky window traps and with a series of H-traps in the close surroundings of the stables. All windows on both farms were equipped with mosquito netting by the farmers (Figure 1).

### 2.2. Window Traps

We applied green plastic meshes (10 × 10 mm mesh width) to the selected windows at each farm. We then applied glue (Sticky-Trap Horsefly Glue) to these plastic meshes to catch any hematophagous insect trying to enter the high-biosecurity pig farm through these windows, in a similar fashion to Müller et al. [33] (Figure 1). On farm A, we covered a total of 8 windows at six different stables, with the plastic mesh (2 windows of 30 × 440 cm, 3 windows of 30 × 360 cm, 1 window of 35 × 360 cm, and 2 windows of 30 × 60 cm, in total 7.5 m^2^). On farm B, we covered eight windows with plastic mesh panels (130 × 30 cm, in total 3.12 m^2^). As this farm only had one stable with windows, eight out of its 24 windows were selected at this farm, all from the same stable.

Since the sticky mesh traps catch both insects and dust, they lose their efficiency over time. We therefore collected samples once every 24 hours and reapplied sticky glue when needed. We removed hematophagous insects (i.e., tabanids (family *Tabanidae*), mosquitos (family *Culicidae*), and stable flies (family *Muscidae*) using forceps and put them into 1.5 mL Eppendorf tubes containing 66% ethanol. On farm A, we collected insects from 19^th^ to 22^nd^ July 2020, while on farm B we collected samples from 26^th^ to 29^th^ July, 2020. Unfortunately, COVID-19 travel restrictions imposed after the first period prevented us from conducting a scheduled second collection period.

### 2.3. Tabanid Traps (H-Traps)

Each farm operated a total of 19 or 20 H-traps around the stables and within the inner fence to reduce influx of hematophagous insects into the stables. These traps were filled with Virkon™ and used infrared light emitted from a large black rubber ball at the bottom to attract horseflies (tabanids). The horseflies were captured in a container on the top of the trap, after which they could be collected. Although these H-traps were designed to attract tabanids, they also collected *S. calcitrans*. We selected five H-traps at each farm and filled them with 66% ethanol instead of Virkon™. We selected the five H-traps to target each corner of the farms. During each farm visit (17th – 21st July at farm A and 25th – 29th July at farm B), we emptied and collected each of the five selected H-traps every 24 hours. After this, the traps were emptied and harvested once a week until 2nd October.

### 2.4. Lab Analysis

The collected insects were stored in 66% ethanol at room temperature at the farm locations and then transferred to the Statens Serum Institut (SSI) in Denmark for further analysis. For logistical reasons, analysis of the samples from farm A was delayed until March 2022. Within the high containment lab, each of the hematophagous insect samples, caught on the plastic meshes or in H-traps, were classified to genus level. After this, we selected and analysed a subsample for the presence of exogenous blood, with “exogenous blood” defined as blood meals not originating from the high biosecurity pig farm itself (i.e., not domestic pig blood, as we assumed this would be from pigs inside the farm). This selection was dependent on how intact or well preserved an insect was, as some insects were damaged or missing their abdomen, and aimed to evenly represent the different collection dates and different traps.

In order to remove surface contamination, we rinsed each collected insect from the window samples in an Eppendorf tube with 2% Virkon S and subsequently 70% ethanol prior to homogenization and analysis. The samples were then individually placed in new 1.5 mL Eppendorf tubes along with 1 mL MagNA Pure Lysis/Binding Buffer (Roche). After this, we added two 3 mm stainless steel beads (Dejay Distribution Ltd.) and homogenized the samples for 3 minutes at 25 Hz using a TissueLyser II (Qiagen). Lastly, we centrifuged the homogenates for 2 minutes at 10.000 RCF and the supernatants were used for DNA extraction with the MagNA Pure 96 system (Roche) as previously described by Olesen et al. [19]. In brief, DNA was purified on the MagNA Pure 96 with the DNA/Viral NA S.V. 2.0 kit following the Viral NA Plasma external lysis S.V. 3.1 protocol.

We screened the samples individually for the presence of a blood meal using two different qPCRs for the detection of the mitochondrial cytochrome b (MT-CYB) gene. One assay identified MT-CYB of mammalian origin and the other assay identified MT-CYB originating specifically from suids.

For the detection of MT-CYB of mammalian origin, we used primer sequences from Andrejevic et al. [34] with minor modifications. The slightly modified primer sequences were 5′ GACGGCCAGTGAAACAGGATCCAACA ACCC 3′ (forward) and 5′ GCTATGACCGGTGTAGTTGTCTGGGTCTCC 3′ (reverse). Briefly, we had shortened both the forward and reverse primers from their 5′ends based on results obtained, using the original primers (38-39 nt primers from Andrejevic et al. [34] and the modified primers (30 nt), during validation of the assay on mammalian samples (data not shown). PCR amplification was performed in a reaction containing 2.5 *μ*L template, 2.5 *μ*L 5 X One-Step RT-PCR Buffer (Qiagen), 1.25 primer-mix (7 pmol/*μ*L), 0.5 *μ*L dNTP, 0.5 *μ*L One-Step RT-PCR Enzyme Mix (Qiagen), 0.5 *μ*L ResoLight (Roche), and 4.75 *μ*L nuclease-free water. Amplification was performed using a CFX Opus Real-Time PCR System (Bio-Rad) with the following conditions: 95°C for 15 min, followed by 40 cycles at 94°C for 1 min, 54°C for 30 sec, and 72°C for 1 min followed by a melting curve protocol. We defined a positive result (indicating mammalian blood present) in this qPCR by the generation of a threshold cycle value (Cq), at which SYBR dye emission appeared above background, within 30 cycles.

For the detection of suid MT-CYB, we used a TaqMan assay, essentially as described by Forth [35]; also using the CFX Opus Real-Time PCR System (BioRad). In brief, we determined a positive result (suid blood present) in this qPCR by identification of a Cq value within 35 cycles.

If mammalian blood was detected (the Cq-value at or below 30 in the MT-CYB mammalian assay), the PCR products generated were selected for sequencing. We purified the fragments using the GeneJET PCR Purification Kit (Thermo Scientific) according to the manufacturer's instructions. Sequencing of the reverse and forward strands was performed using primers (10 *μ*M) and the BigDye Terminator v. 1.1. Cycle Sequencing Kit (Applied BioSystems) according to the manufacturer's instructions using a Veriti™ 96-Well Thermal Cycler (Applied Biosystems) with the following conditions: 96°C for 1 min, followed by 25 cycles at 96°C for 10 sec, 50°C for 20 sec, and 60°C for 3 min. We purified the sequencing products with the SigmaSpin Postreaction columns (Sigma–Aldrich) according to the manufacturer's instructions and performed capillary electrophoresis using the ABI Genetic Analyzer (Applied Biosystems). Lastly, we analysed the results using Sequence Scanner Software v1.0 (Applied BioSystems) and identified the blood meal source using BLAST (https://blast.ncbi.nlm.nih.gov/Blast.cgi).

If porcine blood was detected, either by the suid MT-CYTB assay and/or using Sanger sequencing, we performed further analysis using a TaqMan assay to distinguish between domestic pigs and wild boar. We designed primers to discriminate between the SNP g.299084751C > T in the nuclear receptor subfamily 6 group A (NR6A1) gene located on chromosome 1 [36, 37] using a Custom TaqMan® Assay for SNP genotyping (ThermoFisher). In brief, a *Sus scrofa* NR6A1 sequence (gene ID 100038028) (https://www.ncbi.nlm.nih.gov/gene/100038028) from nt 227 to nt 873 was submitted to the ThermoFisher website and a custom-made primer-probe mix was designed. PCR amplification was performed in a reaction containing 5 *μ*L template, 12.5 *μ*L TaqMan™ Genotyping Master Mix (ThermoFisher), 1.25 *μ*L TaqMan™ Genotyping Assay Mix (ThermoFisher), and 6.25 *μ*L nuclease-free water. Amplification was performed using a CFX Opus Real-Time PCR System (Bio-Rad) with the following conditions: 95°C for 10 min, followed by 40 cycles at 95°C for 15 sec and 60°C for 1 min. We defined a positive result in this qPCR by identification of the threshold cycle value (Cq) at which FAM (SNP g.299084751 C) and/or VIC (SNP g.299084751 T) dye emission appeared above background within 42 cycles.

We tested each sample that yielded a positive result in the mammalian or suid MT-CYTB assays for the presence of ASFV DNA essentially as described by Tignon et al. [38]. We confirmed ASFV positive samples by rerunning the test in triplicate using the virotype ASFV 2.0 (Indical Bioscience) assay according to the manufacturer's instructions. For both assays, we used the CFX Opus Real-Time PCR System (BioRad) and defined a positive result as a Cq value below 42.

After identifying which of the sampled insects carried exogenous blood, we made inquiries with the farm management teams to uncover the closest possible source of this exogenous blood in order to estimate how far the insects had flown while carrying the exogenous blood.

For logistical reasons, H-trap samples from farm B were homogenized and then stored in 1 mL MagNA Pure Lysis/Binding Buffer (Roche) at −80°C, and the H-trap samples from farm A were stored in 66% ethanol for a year. We then pooled the insects according to location, date, trap number, and genus. For practical purposes, we also pooled them according to size, meaning that relatively small insects, such as *Haematopota* spp. and *S. calcitrans* were pooled in groups of five, whereas larger insects such as *Tabanus* spp. were pooled only in groups of two or tested individually. We selected pools based on a proportional representation of each of the various genera/species and across the collection period.

The insects caught in the individual H-traps from farm B were thawed and homogenized in a similar fashion as the window samples, after which we extracted between 0.2 and 0.5 mL of homogenate, depending on the pool size, in order for the final pool to be 1 mL just like the window samples. The H-trap samples from farm A, which had been stored in 66% ethanol at room temperature until this point, were processed in a similar fashion, except that the insects in each pool were first placed into an Eppendorf tube, before 1 mL MagNA Pure Lysis/Binding Buffer and the two stainless steel beads were added. Collection of the supernatants and the subsequent DNA extraction and analyses were performed as described for the window trap samples (see above).

### 2.5. Data Analysis of Exogenous Blood in Insects Caught on Window Traps

We calculated the daily prevalence of exogenous blood meals per insect genus (and per *S. calcitrans*), which was the daily proportion of the total number of samples used for the PCR assays for each individual collection date that were shown to carry exogenous blood as determined by sequencing. As we could not determine whether the samples that tested positive for mammalian blood, but could not be sequenced, were from domestic pigs or originated from mammals outside of the farm, we excluded these samples when calculating the prevalence of exogenous blood meals on the windows. The same applied to samples that were shown (by amplicon sequencing) to have *Sus scrofa* blood but where it was not possible to distinguish between domestic pigs and wild boar.

### 2.6. Data Analysis of Exogenous Blood in Insects Caught in H-Traps

We defined exogenous blood in the same way as for the windows traps. However, since these samples were pooled, we do not know how many individual insects in each of the PCR positive pools were actually positive. Therefore, for each species and for *S. calcitrans* we used an approach as described in https://epitools.ausvet.com.au/ppvariablepoolsize to estimate the prevalence point estimates (+95% CI) of samples containing blood out of the total number of samples of that genus/species in all the pools analysed by PCR.

### 2.7. Data Analysis of Window Insect Numbers and Blood Volume Estimations

For each farm, collection date and each genus caught (species in the case of *S. calcitrans*), we calculated the number of samples caught, as well as the numbers of samples with exogenous blood, for each genus (species in the case of *S. calcitrans*) per day per m^2^ of window surface. We did this by dividing the number of insects caught by the area of the windows covered by window traps (3.12 m^2^ for farm B and 7.14 m^2^ for farm A). Using the average blood meal volume from the literature [39–41] for each genus (and *S. calcitrans*), we attempted to estimate the volume of exogenous blood that is carried by insects onto one m^2^ of window per farm on a given day during the collection period.

## 3. Results

### 3.1. ASFV Detected in Tabanids from H-Traps

From farm A, one pool of five *Haematopota* spp. tested positive for ASFV DNA in both ASFV Taqman assays. Using the assay described by Tignon et al. [38], Cq values were 37, 37.1, and 38, respectively, in three extractions performed from the same pool. Each of the three extracted samples was further tested in triplicate using the ASFV virotype 2.0. with Cq values ranging from 34.1 to 37 in the nine PCR reactions. The pool sample originated from a trap collection emptied on 12^th^ August 2020 and we analysed the pool in June 2022. As all the *Haematopota* spp. samples in the pool were homogenized, we were unable to determine how many insects in the pool were positive for ASFV DNA.

The ASFV DNA positive pool also tested positive using the mammalian MT-CYB assay, but we were unable to obtain sequence information. Using the suid MT-CYB assay the pool had a Cq-value of 40.3 (the threshold for this assay was 35) indicating either a very low amount of swine blood within it or that the blood was degraded. Due to this, the pool was not tested further to determine whether the blood originated from wild boar or domestic pigs, as this amount of blood would be below the limit of detection for the SNP genotyping assay. According to the Lithuanian veterinary services (December 2020, data extracted from state veterinary maps), there were no reported ASF-outbreaks in domestic pigs in the area, but two hunted wild boar, both within a 10 km radius from the farms, tested positive for the virus between the start of our experiment through to 2^nd^ November 2020. The first wild boar was hunted 8.6 km from the farm on 7^th^ September 2020, and the second wild boar was hunted 7.3 km from the farm on 8^th^ October 2020. The positive wild boar cases indicate the presence of ASFV in the vicinity of the farm. Despite the time interval between our collection of an ASFV positive sample and the detection of the wild boar cases, we do find it likely that the virus had been circulating in wild boar for some time.

### 3.2. Insects and Blood Meals Detected following Capture on the Window Traps

On farm A, we caught 148 insects from the window traps, including 40 *Aedes* spp., 26 *Anopheles* spp., 5 *Culex* spp., 62 *Haematopota* spp., and 15 *Tabanus* spp. in 4 days. From these, we performed PCR analysis on 147 samples to detect mammalian blood and also specifically blood of suid origin. Using the suid MT-CYB qPCR, none of the samples tested positive (no Cq value or Cq value above 35). Twenty samples tested positive in the mammalian MT-CYB assay (13.6%) and were analysed by sequencing to identify the host species. The results showed that two *Haematopota* spp. samples carried *Bos taurus* (cattle) blood and one *Aedes* spp. sample carried *Capreolus capreolus* (Roe deer) blood, while five samples carried *Sus scrofa* blood. Using the Custom TaqMan® Assay for SNP genotyping, it was found that one sample carried domestic pig blood as it yielded a signal for VIC (SNP g.299084751 T) only, while the remaining four samples did not yield a signal for either of the two SNPs. Hence, these could be of either wild boar or domestic pig origin. Twelve samples were PCR positive for mammalian blood, but were too degraded for further sequencing to identify the host species. The remaining 127 samples did not test positive for mammalian blood (Table 1).

Excluding the samples that only tested PCR positive for mammalian blood but could not be sequenced, as well as the samples that were identified as containing blood from *Sus scrofa*, but could not be further distinguished, only three samples out of 147 (2.0%) carried blood meals, which certainly originated from outside the farm (Table 1).

On farm B, we caught 495 insects from the window traps, including 84 *Aedes* spp., 151 *Anopheles* spp., 59 *Culex* spp., 113 *Haematopota* spp., and 88 *S. calcitrans* in 4 days. From these, we performed PCR analysis on 249 samples to detect mammalian blood and identify blood of the suid origin. Seven samples tested positive for suid blood using the suid MT-CYB assay, while 4 samples tested positive in the mammalian MT-CYB assay. Sequencing of the 11 PCR positive samples (4.4%) showed that two *Haematopota* spp. and two *S. calcitrans* samples carried *Bos taurus* (cattle) blood, while the seven samples that tested positive in the suid MT-CYB assay did indeed carry blood of the suid origin. When using the Custom TaqMan® Assay for SNP genotyping on these seven samples, it was indicated that six of the seven samples carried domestic pig blood as they yielded a signal for VIC (SNP g.299084751 T) only, while the remaining sample did not yield a signal for either of the two SNPs. Hence, this blood meal could be of either wild boar or domestic pig origin. The remaining 238 samples tested did not give a positive result for mammalian blood (Table 1).

Excluding the samples that only tested PCR positive for mammalian blood but could not be sequenced, as well as the samples that were identified as containing blood from *Sus scrofa*, but could not be further distinguished, we found 4 samples out of 249 (1.6%), which carried blood meals that certainly originated from outside the farm (Table 1).

**Figure 2:**
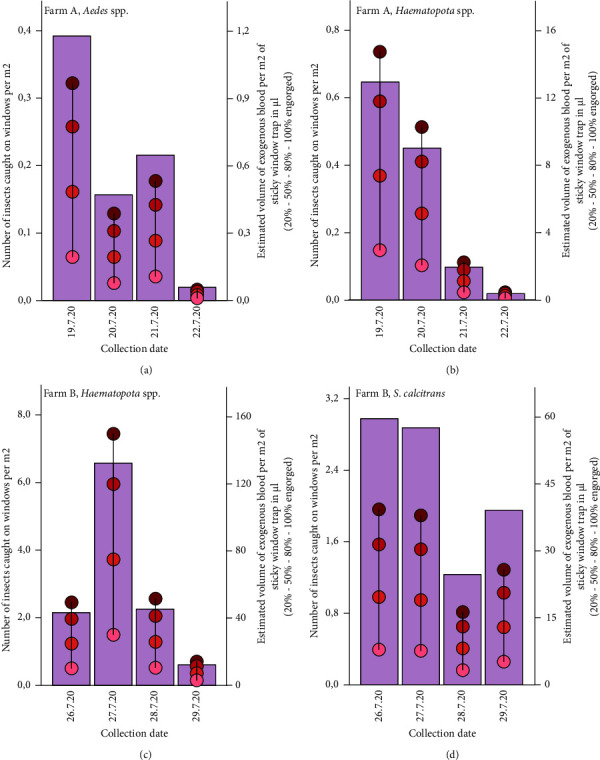
The daily number of insects caught, adjusted to 1 m^2^ of window trap (bars and left *Y*-axis) and four estimates of the blood meal volumes they would carry (dots and the vertical drop line, indicating 100%–80% - 50%–20% engorged, scale on right *Y*-axis). Note that *Y*-axes scales vary within and between graphs.

In order to make estimates of the volume of exogenous blood in insects collected per m^2^ of window surface per day, as well as estimates of the volume of blood in the H-traps, we used the average blood meal volume for each genus (and *S. calcitrans*) we caught on both the window traps and H-traps. Based on the average values of blood meal volumes in the published literature, *Haematopota* spp. has an average blood meal volume of 22.8 *μ*l [39], *S. calcitrans* (combined mean of females and males) 13.2 *μ*l [41] and *Aedes* spp. 2.47 *μ*l [40]. Hematophagous insects with blood that are attracted to a host or a pig farm can be either partially or fully engorged [42, 43]. For the estimations of the total blood volumes carried by insects attempting to enter the farm or present in the farm surroundings, we therefore assumed that they would carry an amount of blood equivalent to 20%, 50%, 80%, or 100% of the average blood meal volumes for each genus (and *S. calcitrans*) (Figure 2). It should be noted however that these estimates should not be interpreted as exact quantitative measurements of potential infection pressure or an introduction risk, as the system is highly variable and uncontrollable. Rather, these estimates should only be used to provide insights into whether the amount of (potentially infectious) exogenous blood constitutes an insignificant or non-negligible risk of virus introduction.

On farm A, all 10 stables had windows of varying sizes and numbers. The total window surface was 247.4 m^2^, of which we surveyed 7.14 m^2^ (2.9%), distributed between 8 windows on 6 stables.

On farm B, only one out of 35 stables had windows, whereas the remaining 34 stables were solely lit with artificial lighting and the only entry was through a door from the interior hallway. The stable with windows had a total window surface area of 6.32 m^2^, of which we surveyed 3.12 m^2^ (49.4%) distributed between 8 windows.

We observed a marked daily variation in the number of insects caught on the window traps, as well as higher numbers in farm B compared to farm A (Figure 2). On 21^st^ July 2020 and 22^nd^ July 2020, the weather was cloudy and rainy (Table 1), which likely negatively influenced the catch numbers of horseflies.

### 3.3. Insects and Blood Meals Detected in the H-Traps

On farm A, we caught 1613 insects in the five selected H-traps. Of these, we caught 1235 *Haematopota* spp., 317 *Tabanus* spp., and 61 *Stomoxys calcitrans*. In total, 82 pools were visually of a quality suitable for PCR analyses, which included 326 out of 1613 insects (20.2%). However due to logistical constraints, we selected 33 pools for PCR analysis (made from 129 insects). Out of these selected pools, 6 pools (18.2% of analysed pools) were PCR positive for mammalian blood and so analysed by sequencing. In these 6 pools, one pool of *S. calcitrans* (*N* = 5) was shown to contain *Bos taurus* (cattle) blood, while 1 pool of *S. calcitrans* (*N* = 2) contained *Canis lupus familiaris* (domestic dog) blood. Two pools of *Haematopota* spp. (*N* = 5, 5, respectively) had *Bos taurus* (cattle) blood, while 2 pools of *Haematopota* spp. (*N* = 5, 5, respectively) could not be sequenced.

The estimated prevalence of individual blood fed *Haematopota* spp. at farm A was 3.3% [0.8%, 8.3%] assuming the probability of catching a blood fed insect among other insects is random. For *S. calcitrans*, estimated prevalence for blood fed insect samples was 5.5% [0.9%, 16.1%].

On farm B, we caught 1963 insects in five selected H-traps. Of these, we caught 1744 *Haematopota* spp., 197 *Tabanus* spp., 3 *Chrysops* spp., 3 *Hybomitra* spp., 1 *Atylotus* spp., 12 *S. calcitrans*, 1 *Anopheles* spp., and 2 *Culex* spp. After discarding insects unsuitable for PCR, we ended up with 440 pools (1753 out of 1963 insects, or 89.3%). Of these pools, we selected 147 pools for PCR analysis (520 out of 1753 insects, or 29.7%). Four pools (2.7% of analysed pools) tested positive for mammalian blood and were analysed by sequencing. Of these four pools, one pool of *Tabanus* spp. (*N* = 2) had *Sus scrofa blood*, while the other three pools, from *Haematopota* spp. (*N* = 1, 5, 2, respectively), could not be sequenced, probably due to degradation. Since we cannot know if these pools contained domestic pig blood, we excluded them from the calculations of the prevalence point estimate (+95% CI) for blood fed samples.

The estimated proportion of exogenous blood from farm A was applied to farm B, as for the latter farm the quality of the H-trap pools with mammalian blood was not sufficient to sequence the samples. Although host-availability between the farms can vary, our results from the window traps do indicate that certain hosts, other than domestic pigs, were present in the surrounding environment.

**Figure 3:**
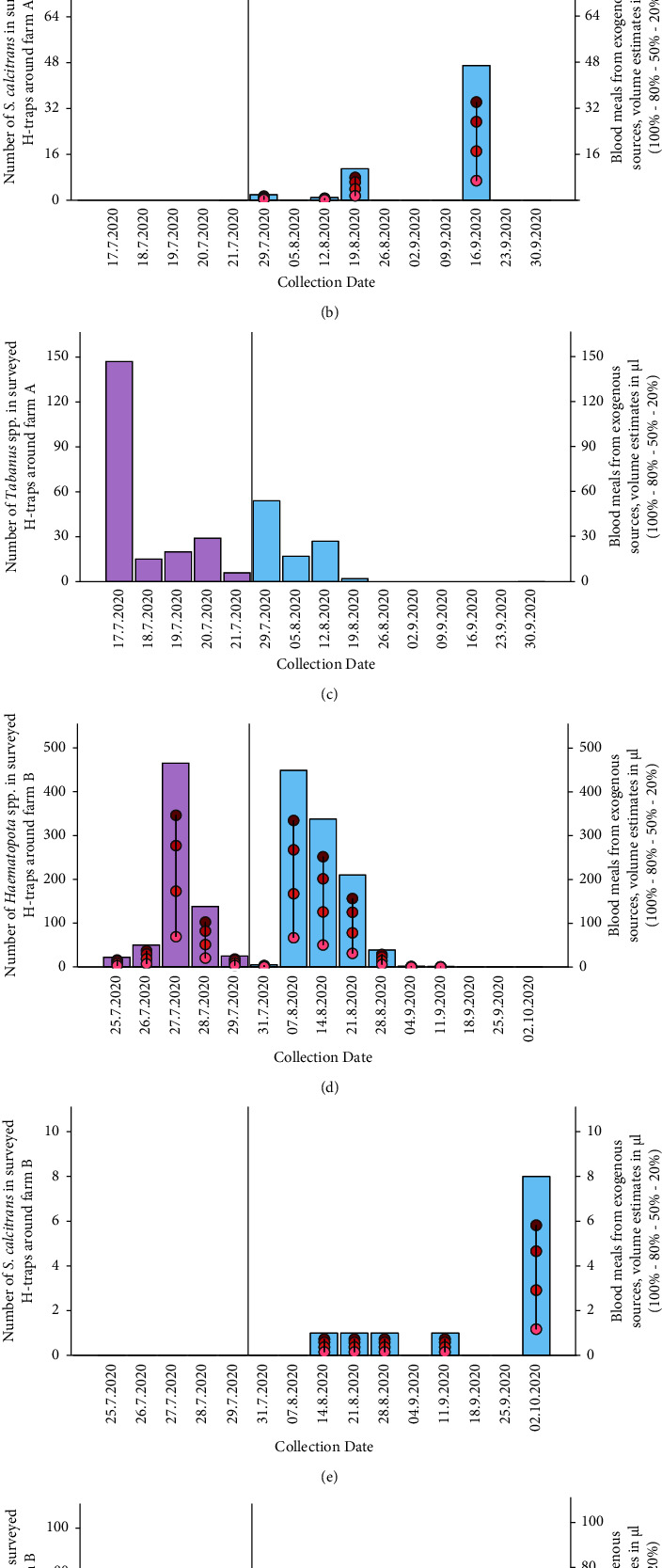
The number of insects caught in all 5 H-traps combined, for each collection date on each farm. Purple bars indicate daily collections and blue bars indicate weekly collections, scale on left *Y*-axis. Note that at farm A there were only 2 days in between the last daily collection and first weekly collection, whereas at farm B this was 8 days. The dots and drop line indicate estimates of the blood meal volumes they would carry (dots and vertical drop line, indicating 100%–80% - 50%–20% engorged, scale on right *Y*-axis). Other genera such as *Atylotus* spp. (*N* = 1), *Chrysops* spp. (*N* = 3), and *Hybomitra* spp. (3) are not presented due to their low numbers. Note that *Y*-axes scales vary between graphs.

*Haematopota* spp. had the highest abundance in traps on the pig farms and was estimated to carry the largest volume of exogenous blood throughout the season of the species we caught (Figure 3). *S. calcitrans* sporadically appeared in our H-traps towards the end of the collection period and was occasionally carrying exogenous blood although it must be stressed that the H-traps are not designed to catch *S. calcitrans*. *Tabanus* spp., while present in considerable numbers (317 at farm A and 197 at farm B), they did not carry any exogenous blood in our H-traps.

From our daily collections (Figure 3, purple bars), we observed significant variation in the number of insects caught per day (Figure 3). On two days, 21^st^ July 2020 and 22^nd^ July 2020, when low numbers of insects were caught, the weather was cloudy and rainy (Table 1).

We found no significant difference in the number of insects caught between the five traps on each of the two farms, neither for each individual genus nor for all genera combined, across the collection period. Appendix I shows the median numbers of all insects combined per trap per collection date for each farm.

### 3.4. Flight Distance of Exogenous Blood Meals Found in Both H-Traps and Window Traps

For farm A, after inquiry, we found that the closest reported source for the bovine blood meals was approximately 700 m from the farm. There were no dogs present on the farm property, meaning that the one dog blood meal (*Canis lupus familiaris*) must be considered exogenous, but we are unable to deduce the distance this blood meal must have travelled. The same applies to the roe deer blood meal (*Capreolus capreolus*), which could have originated from anywhere beyond the farm's outer fencing and areas to which wild boar also have access.

For farm B, after inquiry, we found that the closest reported source for the bovine blood meals was 2500 m from the sampling site. Of this, 1750 m spanned coniferous forest, while the remaining 750 m was across open agricultural fields. It should be noted, however, that adjacent to the bovine source, there was a road leading through the forest directly towards the high-biosecurity pig farm, which the insects could potentially have used instead of flying through the actual forest.

## 4. Discussion

Our finding of ASFV DNA-positive blood in one of the pools from our H-traps, containing five *Haematopota* spp., indicates that ASFV was circulating in the environment closely surrounding the high-biosecurity pig farms, inside the protective wildlife fences. Although we did not find any ASFV DNA-positive insects on the window traps, we did find blood fed *Haematopota* spp. carrying blood meals from exogenous sources on the windows, believed to originate from a source 2.5 km away. This indicates that *Haematopota* spp. is still attracted to pig farms while carrying a blood meal. Their high abundance during their seasonal peak, with several thousand flying around a high-biosecurity pig farm, indicates that *Haematopota* spp. can carry non-negligible volumes of blood into high-biosecurity pig farms, and the finding of one positive pool indicates that some of this blood can contain ASFV obtained from infected wild boar. Olesen et al. [7] calculated that a single stable fly (that likely carries a significantly smaller volume of blood compared to a tabanid), after feeding on pig blood carrying a realistic titre of ASFV, could theoretically carry a high enough level of virus (3.8–4 log_10_ TCID_50_) to establish an infection through oral inoculation. Howey et al. [44] showed that, out of several possible inoculation pathways, oral inoculation requires the highest viral dose to establish infection. We were not able to analyse whether the ASFV DNA-positive blood we found was infectious or not and what the viral load was. However, given the high viral load of ASFV found in blood [19, 25], the relatively large blood meal size of tabanids [39] and the dose required to cause an infection, it seems likely that they can carry sufficient blood to contribute to the risk of ASFV spreading from wild boar to domestic pigs within high biosecurity farms when ASFV-infected wild boar are present in the area surrounding these farms.

Although we did not catch any *S. calcitrans* carrying blood meals containing ASFV, we did catch *S. calcitrans* carrying *Sus scrofa* blood from domestic pigs and, in one case, *Sus scrofa* (either domestic pig or wild boar) origin, on the windows. Additionally, we caught *S. calcitrans* attempting to enter the pig farm while carrying bovine blood meals believed to originate from outside sources up to 2.5 km away. This indicates that this insect species could also contribute to the introduction of blood containing ASFV into high-biosecurity pig farms, when there are ASFV-positive wild boars present in the vicinity. Turčinavičienė et al. [32] reported a peak in *S. calcitrans* abundance towards the second half of October, in line with our catches of *S. calcitrans*. However, the sporadic appearance of *S. calcitrans* observed in our study could likely have been influenced by our use of H-traps, which are not designed to target *S. calcitrans* (or mosquitos).

We also found *Aedes* spp. carrying roe deer blood (*Capreolus capreolus*) on the window traps. Previous studies showed that *Aedes* spp. do feed on domestic pigs and are generally opportunistic mammalophilic feeders, suggesting they would also feed on wild boar when available. This indicates that *Aedes* spp. may also contribute to the introduction of blood containing ASFV into high-biosecurity pig farms, when there are ASFV-positive wild boars present within the vicinity. Lastly, we found that *Tabanus* spp. carried domestic pig blood, as well as *Sus scrofa* blood, that may potentially be of wild boar origin onto the window traps. Although we have no evidence for *Tabanus* spp. carrying blood from outside sources into the farms, the fact that they carried a blood meal onto the window traps from outside, as well as their reputation as aggressive feeders and strong flyers, indicates they could also potentially be involved in carrying blood containing ASFV into high-biosecurity pig farms in Lithuania. Weiner and Hansens [45] showed that certain North American *Tabanus* spp. have a strong feeding preference for domestic pigs. Baldacchino et al. [29] found that *Tabanus bromius* feeds on wild boar, while Tidwell et al. [46] found that several *Tabanus* spp. are able to mechanically transmit classical swine fever virus. While feeding preferences of hematophagous insects vary between species, and data are lacking regarding the species-specific host preferences of many tabanids, they are regarded as opportunistic predators that feed on any large available mammalian host [29, 47].

The proportion of insects with exogenous blood meals was 2% and 1.6% in the window samples from farm A and farm B, respectively, and 12.1% in H-traps pools from farm A. However, we only surveyed a subsample of the entire farms, i.e. 2.9% of the window surface on farm A and 49.4% on farm B, as well as approximately 25% of H-traps on each farm. Furthermore, the results from the H-traps can be used as a proxy for the numbers of insects in the surroundings, but clearly only catch a fraction of the total number of insects flying around. Still, combining these numbers with the estimated average blood meal sizes of the various genera/species analysed, indicates that blood volumes that could be large enough to carry a significant level of ASFV into the farms was being transported by these insects. For example, if we extrapolate our findings from the window traps on farm A on a warm day (19^th^ July 2020) to the entire farm's window surface (247.4 m^2^, of which we surveyed 7.1 m^2^), around 1144 *Haematopota* spp., 693 *Aedes* spp., (and 277 *Tabanus* spp., which did not carry exogenous blood), could attempt to enter the farm on a single day. If 2% of those were carrying exogenous blood that would be approximately 23 *Haematopota* spp. (20%–100% engorged would equal 105 *μ*l to 524 *μ*l of blood, based on Hollander and Wright [39] and 14 *Aedes* spp. (20%–100% engorged would equal 7 *μ*l–34 *μ*l based on Ogunrinade [40]). If we do the same for *Haematopota* spp. from our H-trap collections, extrapolated to the total number of H-traps around farm A and as a sum for the entire collection period, the H-traps would catch around 4940 *Haematopota* spp. If we then apply our estimate of exogenous blood prevalence, as well as average blood meal volume, there would be 163 *Haematopota* spp. throughout the collection period, carrying between 744–3716 *μ*l (20%–100% engorged) of exogenous blood. Even though the blood will not necessarily go into the stables, it is carried into the fenced area of the farm, which is the same area that a potentially contaminated truck would gain access to.

Our results indicate that hematophagous insects (*Haematopota* spp. and *S. calcitrans*) are probably capable of carrying blood meals from sources at least 2.5 km away into high-biosecurity pig farms. This distance is significantly longer than reported distances for semi-/fully engorged insects in previous studies, namely 600 m for *Tabanus bromius* [29] or 170 m for mosquitos [28]. It should be noted that near farm B, there was a meat processing facility present 350 m from the pig farm, which processed beef. However, access to the carcasses that arrived at this facility was very restricted for any flying insects, and as these carcasses were already butchered and drained of blood, it is very unlikely that this could have been the source of the bovine blood meals found in the insects. Assuming that the longer flying-range of blood fed insects indicated here is correct, this means that any ASFV infected wild boar or domestic pig within 2.5 km of a farm can act as a source of blood containing ASFV for hematophagous insects, which can then be introduced into a farm with uninfected pigs. This finding correlates with the findings of Boklund et al. [17]; where proximity of ASF outbreaks in domestic farms was found to be a significant risk factor for commercial farms, and additionally that wild boar cases were a risk for backyard farms in Romania. However, Pautienius et al. [13] reported no correlation between wild boar density and the number of ASF outbreaks in domestic pigs in Lithuania.

After ASF prevalence peaked amongst wild boar in 2017 [13] the prevalence gradually decreased along with the reduced wild boar population between 2016 and 2020 [15]. This made it less likely for us to catch any (ASFV-positive) wild boar blood meals. Between 1^st^ September 2019 and 31^st^ August 2020, only one ASF outbreak in a commercial farm and two ASF outbreaks in backyard farms were reported in Lithuania, while there were 306 reported cases of infected wild boar, of which 190 were hunted and 116 were found dead [14].

The results of our PCR analysis allowed several different interpretations with respect to which blood meal were regarded as exogenous. Our results showed a notable difference in the percentage of insects that we could successfully analyse by sequencing. For the window traps from farm A and farm B, 8/20 (40%) and 11/11 (100%) of insect samples that were PCR positive for mammalian blood could be successfully sequenced, respectively. For the H-traps from farm A and farm B, we successfully obtained sequence information for 4/6 (83%) and 1/4 (25%) of the pools that were PCR positive for mammalian blood, respectively. These differences are likely due to the different time intervals between collection and analysis as well as different storage methods applied due to logistical circumstances. For these reasons, we decided to apply the most conservative interpretation of our data and only included those blood meals that were certainly exogenous for our prevalence and blood meal volume estimates. When we compared the percentage of exogenous blood meals out of the total number of PCR positive blood meals for each farm and trap type, we found that 3/20 (15%) of window trapped insects compared to 4(+1)/6 (83%) of H-trap pools from farm A and 4/11 (36%) of window trapped insects compared to 0% of H-trap pools from farm B. The (+1) exogenous blood sample was from the ASFV DNA positive pool, which could not be sequenced, but was assumed to be exogenous, as there was no ASF outbreak on the farm. From these different percentages, we argue that some of the mammalian blood PCR positive window insects from farm A and mammalian blood PCR positive H-trap pools from farm B would have likely carried exogenous blood.

We caught significantly fewer insects on the windows of farm A (148) than on farm B (495). Part of this difference can be attributed to poor weather conditions on farm A on 21^st^ and 22^nd^ of July, with rain (showers) and wind speeds of over 5 m/s, the latter is known to negatively affect the activity of hematophagous insects [48]. It is also possible that the lack of windows on farm B, where only 1 out of 35 stables had windows, contributes to an accumulating effect as insects may circle around the farm until they find an entry opportunity. Furthermore, the windows on both farms had screens, so insect numbers on the windows could be inflated due to flies bouncing off a screened (non-sticky) window and eventually finding their way onto the sticky mesh. Conversely, H-traps located around the farm could attract an insect on its way onto the farm, therefore possibly deflating the likelihood that an insect would end up on the sticky window mesh. During collection, we saw several large horseflies, presumably *Tabanus* spp. based on their size, fly against the sticky window traps but bounce off immediately or freeing themselves after some struggling. This is likely due to their large size, consequently making them stronger flyers.

Insects caught on the window traps, were interpreted as “insects trying to enter the farm.” However, we cannot state for certain that the insects were actually trying to get into the interior of the pig stable, rather than just being attracted to the sticky window mesh itself or just trying to find a resting spot on the mesh. From our own observations, however, many of the tabanids were flying onto the window traps with such a high speed that it was unlikely that they were trying to land on them. For *S. calcitrans* and mosquitos, it seems more likely that they are attracted to the odours emitted from inside the farm rather than to the sticky window mesh.

For the qPCR assays, it should be noted that both MT-CYB assays yielded Cq-values above the thresholds for several samples in the mammalian MT-CYB assay and the suid MT-CYB assay, respectively. These samples were regarded as not containing any blood meals of the mammalian and suid origin, even though we cannot rule that they did contain a low amount of mammalian or suid blood. We based the thresholds for the MT-CYB assays on prior validation using templates of the mammalian, avian, and invertebrate origin (mammalian MT-CYB assay-data not shown) and of the different mammalian origin (suid MT-CYB assay-data not shown). However, some insects containing a blood meal could be missed using these cut-offs. Still, using the two MT-CYB assays clearly allows for a straightforward and fast initial screening for the presence of blood meals of the mammalian and suid origin in insects using qPCR. For some samples containing *Sus scrofa* blood, we were able to identify these as being of probable domestic pig origin using the assay designed to distinguish the SNP g.299084751 C > T in the *NR6A1* gene. Hence, using this assay, it was found that seven samples containing *Sus scrofa* DNA were of domestic pig origin as these samples tested positive for the T allele only. The SNP g.299084751 is associated with the number of thoracic and lumbar vertebrae in suids [36, 49] and studies show that the majority of domestic pigs are homozygous for the g.299084751 T while wild boar are heterozygous or homozygous for the wild type allele g.299084751 C [36]. These findings by Kaltenbrunner et al. [36] are in line with the results obtained during validation of this assay in our laboratory with blood samples originating from wild boar and domestic pigs (data not shown).

Our study provides quantitative estimates of the numbers of hematophagous insects attempting to enter high-biosecurity pig farms with uninfected animals, the percentage of (exogenous) blood-carrying insects, and the volumes of blood they are carrying. We demonstrated evidence of blood containing ASFV DNA being carried by hematophagous insects from the surrounding environment directly into these farms, and the results indicated an expansion of the range that hematophagous insects are known to carry blood meals. Our findings confirmed that semi-/fully engorged insects are still attracted to high-biosecurity pig farms and that these insects are a potential source of introduction of blood containing ASFV. The low proportion of insects carrying exogenous blood, when applied to the large numbers of hematophagous insects throughout the vector season, can introduce a considerable volume of exogenous blood directly into pig stables. On high-biosecurity pig farms, transmission pathways via contaminated equipment, feed, workers, or from arriving trucks are mitigated by strict decontamination and quarantine measures. However, hematophagous insects are specifically attracted to the pigs, inside the stables, and could directly introduce blood containing ASFV to them. While the ingested volume of blood is not equal to the volume of blood potentially transferred via subsequent feeding, the ingestion of an insect by an uninfected pig would effectively transfer the entire blood meal into the body of the pig in sufficient volumes to cause an infection through oral inoculation [7]. Mitigating actions against hematophagous insects are often lacking or limited although such actions are reportedly increasing. Our study shows that hematophagous insects can constitute an additional route of virus introduction, which unlike other known transmission pathways, also fits with the observed seasonal pattern of outbreaks on commercial pig farms. The volumes of exogenous blood found in insects in this study, as well as the carriage of blood containing ASFV directly from outside a high-biosecurity farm show that hematophagous insects should not be regarded as a negligible route for introduction of ASFV into high biosecurity farms.

## Figures and Tables

**Table 1:** Overview of blood meal origins from the window trap samples from both farms, for each collection date.

Locations	Date	Samples	Samples analysed by PCR	Samples with mammalian blood^1^	Samples with suid blood^2^	Samples with domestic pig blood^3^	Samples with cow blood (Sanger sequencing)	Samples with deer blood (Sanger sequencing)	Samples with nonspecified mammalian blood^4^	Proportion of samples with exogenous blood, cow and deer regarded as exogenous (%)	Proportion of samples with exogenous blood, cow and deer and nonspecified regarded as exogenous (%)	Proportion of samples with mammalian blood (%)	Average temperature (°C)	Average wind speed (m/s)	Weather description^5^
Farm A	19th July	75	74	16	5	1	1	1	9	2.7	14.9	21.6	21.5	1.4	Fair
Farm A	20^th^ July	41	41	4	0	0	1	0	3	2.4	9.8	9.8	21	2.5	Fair with light rain
Farm A	21^st^ July	30	30	0	0	0	0	0	0	0.0	0.0	0.0	17.5	5	Mostly/partly cloudy with light rain
Farm A	22^nd^ July	2	2	0	0	0	0	0	0	0.0	0.0	0.0	13.7	5.5	Mostly cloudy with rain/showers
Farm A	Total	148	147	20	5	1	2	1	12						
Farm B	26^th^ July	88	84	2	0	0	2	0	0	2.4	2.4	2.4	18.8	1.3	Fair
Farm B	27^th^ July	159	102	7	6	6	1	0	0	1.0	1.0	6.9	21.4	1.9	Fair
Farm B	28^th^ July	128	38	1	0	0	1	0	0	2.6	2.6	2.6	20.1	2.2	Mixed cloud and some rain
Farm B	29^th^ July	120	25	1	1	0	0	0	0	0.0	0.0	4.0	19	4.4	Partly cloudy
Farm B	Total	495	249	11	7	6	4	0	0						

^1^Only samples positive for mammalian blood (i.e., MT-CYB Cq < 30) or that tested positive in the suid MT-CYTB (i.e., MT-CYB Cq < 35) assay were sequenced. ^2^This includes samples sequenced as *Sus scrofa* blood, as well as PCR positive samples from the suid assay (MT-CYB Cq < 35). ^3^Only samples positive for *Suidae* blood in the suid MT-CYTB assay or identified as suid using sequencing were further analysed with the SNP-specific custom-made Taqman assay (Cq < 42). No window trap samples tested positive for wild boar blood. ^4^Only samples positive for mammalian blood (i.e., MT-CYB Cq < 30) that could not be sequenced. ^5^Fair weather is generally pleasant weather conditions, with no precipitation and no extremes of wind or temperature, with less than 3/8^th^ opaque cloud coverage. Light rain is defined as <2.5 mm of precipitation per hour. Partly clouded is defined as 3/8^th^ to 5/8^th^ opaque cloud cover while mostly clouded is defined as 6/8^th^ to 7/8^th^ opaque clouded cover. Showers are defined as sudden precipitation with rapid changes of intensity. All definitions are according to the US National Weather Service (http://www.weather.gov).

## Data Availability

The data used in this study are available upon reasonable request from the corresponding author.

## Supplementary Materials

Supplementary Materials Histograms displaying the median number of all insects combined per H-trap for each collection date on each farm can be found online in the Supporting Information section at the end of this article.
