# Supplementary figures and images for: Potential for Introduction of African Swine Fever Virus into High-Biosecurity Pig Farms by Flying Hematophagous Insects
Source: Transbound Emerg Dis. 2023 Apr 12;2023:8787621. doi: 10.1155/2023/8787621 (PMC12017163; doi:10.1155/2023/8787621)

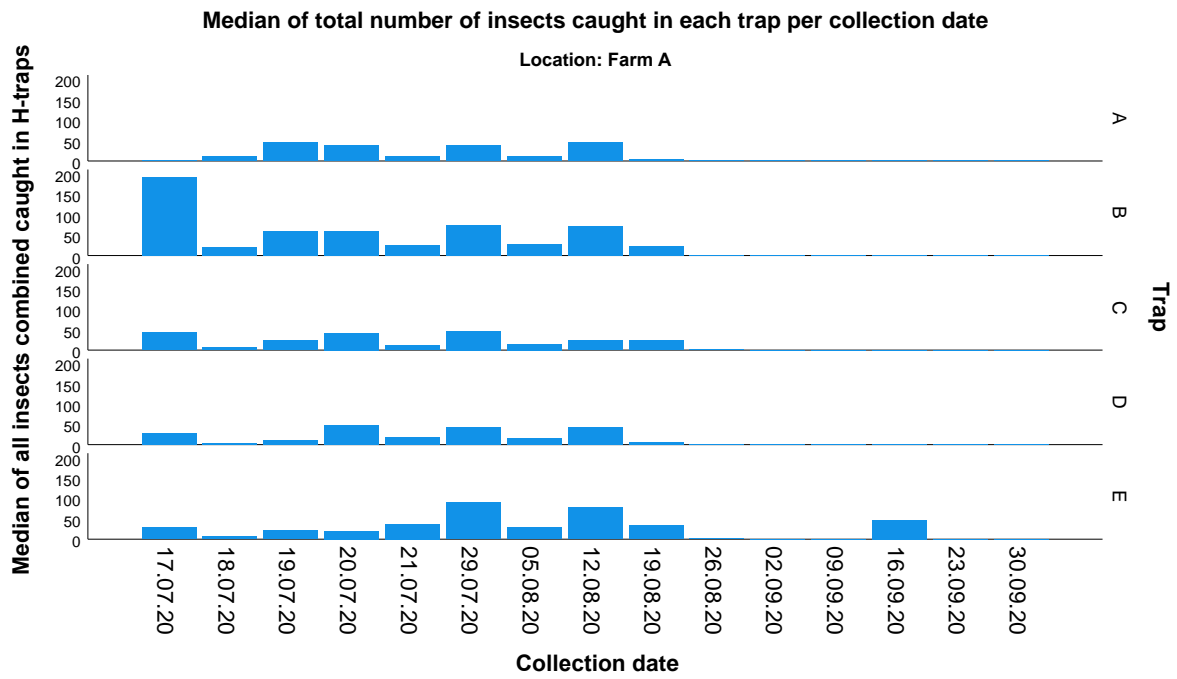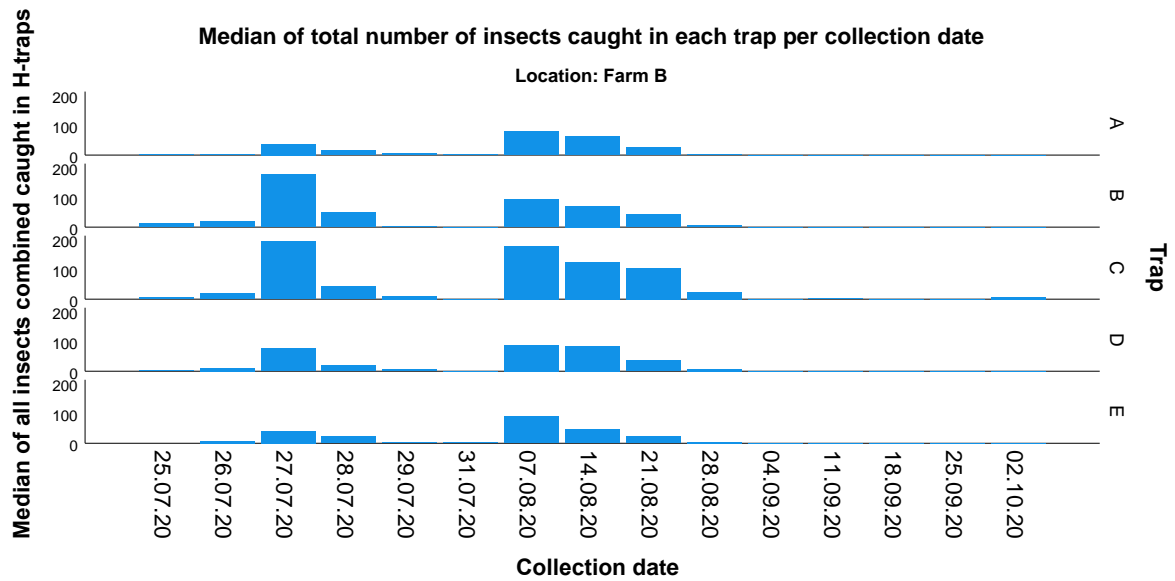

Supplement: Supplementary Materials — Histograms displaying the median number of all insects combined per H-trap for each collection date on each farm can be found online in the Supporting Information section at the end of this article. [file 8787621.f1.pdf]
